# A novel method for identifying disease associated protein complexes based on functional similarity protein complex networks

**DOI:** 10.1186/s13015-015-0044-6

**Published:** 2015-04-28

**Authors:** Duc-Hau Le

**Affiliations:** School of Computer Science and Engineering, Water Resources University, 175 Tay Son, Dong Da, Hanoi, Vietnam

**Keywords:** Disease protein complex, Functional similarity protein complex network, Neighborhood-based algorithm, Prostate cancer

## Abstract

**Background:**

Protein complexes formed by non-covalent interaction among proteins play important roles in cellular functions. Computational and purification methods have been used to identify many protein complexes and their cellular functions. However, their roles in terms of causing disease have not been well discovered yet. There exist only a few studies for the identification of disease-associated protein complexes. However, they mostly utilize complicated heterogeneous networks which are constructed based on an out-of-date database of phenotype similarity network collected from literature. In addition, they only apply for diseases for which tissue-specific data exist.

**Methods:**

In this study, we propose a method to identify novel disease-protein complex associations. First, we introduce a framework to construct functional similarity protein complex networks where two protein complexes are functionally connected by either shared protein elements, shared annotating GO terms or based on protein interactions between elements in each protein complex. Second, we propose a simple but effective neighborhood-based algorithm, which yields a local similarity measure, to rank disease candidate protein complexes.

**Results:**

Comparing the predictive performance of our proposed algorithm with that of two state-of-the-art network propagation algorithms including one we used in our previous study, we found that it performed statistically significantly better than that of these two algorithms for all the constructed functional similarity protein complex networks. In addition, it ran about 32 times faster than these two algorithms. Moreover, our proposed method always achieved high performance in terms of AUC values irrespective of the ways to construct the functional similarity protein complex networks and the used algorithms. The performance of our method was also higher than that reported in some existing methods which were based on complicated heterogeneous networks. Finally, we also tested our method with prostate cancer and selected the top 100 highly ranked candidate protein complexes. Interestingly, 69 of them were evidenced since at least one of their protein elements are known to be associated with prostate cancer.

**Conclusions:**

Our proposed method, including the framework to construct functional similarity protein complex networks and the neighborhood-based algorithm on these networks, could be used for identification of novel disease-protein complex associations.

**Electronic supplementary material:**

The online version of this article (doi:10.1186/s13015-015-0044-6) contains supplementary material, which is available to authorized users.

## Background

Protein complexes are formed by non-covalent interactions among proteins and have specific biological functions. These protein complexes and their cellular functions have been concurrently identified by a number of methods based on protein interaction networks [1-3] and affinity purification-mass spectrometry experiments [4]. However, their particular roles in terms of causing disease have not yet been well-determined. Indeed, all protein complexes in a most updated database of protein complexes CORUM [5] have been well functionally annotated and categorized; however, few of them have a comment on their association with diseases.

It is shown that interactions among proteins forming protein complexes do not only provide a better understanding of cellular functions, but also improve our understanding about human diseases [6-9]. A number of studies have shown the association between protein complexes and specific diseases. For instance, a protein complex of SCRIB, NOS1AP and VANGL1 is associated with breast cancer progression [10], TWIST/Mi2/NuRD protein complex has an essential role in cancer metastasis [11], aberrant protein complex consisting of prostaglandin-d-synthase (PDS) and transthyretin (TTR) is a biomarker of Alzheimer’s disease [12]. In addition, past studies show that mutations in multiple proteins that form a protein complex may lead to the same disease phenotype. Therefore, protein complexes can be used to predict phenotypic effects of gene mutation and identify human disease genes [8]. Some early studies made use of protein complexes to predict novel disease genes [13,14]. However, they did not use actual protein complexes, but those simply assembled by neighboring proteins [13] or generated from densely connected subsets of ranked proteins [14]. In other words, the formation of such protein complexes was mainly based on topological properties rather than functional similarities of their protein elements. In addition, biological relationships between protein complexes were also omitted in those studies. Considering an observation that if two protein complexes have biological relationships (e.g., they share a number of common protein elements or their protein elements are highly physically connected), the mutations of genes in one protein complex can lead to same or similar phenotypes of the other protein complex [15], the functional interaction between protein complexes can play an important role in predicting phenotypic effects of gene mutation. Indeed, a recent study [16] used a heterogeneous network consisting of a global protein complex network layer and phenotype similarity network layer to predict novel disease phenotype-gene associations. In that study, the protein complex network layer was constructed using existing human protein complexes and a human protein interaction network. Then, a network propagation algorithm was applied on the heterogeneous network to prioritize candidate genes. Ultimately, they reported that their method outperformed other methods which were solely based on the human protein interaction network and phenotype similarity network for the prediction of novel disease phenotype-gene associations [17,18]. Taken together, these studies indicate that the protein complexes can be used to improve predictability of novel disease phenotype-gene associations. However, identification of novel direct disease-protein complex associations has not yet been well-focused. Indeed, only a few studies have directly focused on this problem recently [19-22]. For instance, study [19] used a complicated heterogeneous network including three layers (i.e., a phenotypic similarity network layer, a tissue-specific protein interaction network layer, and a protein complex membership layer), and then applied a network propagation algorithm on that network to discover disease-associated protein complexes. However, the phenotype similarity network was collected from a relatively old published study [23]; therefore it is not up-to-date. In addition, they were also limited in the prediction of disease of which a tissue-specific protein interaction network [19] or gene expression data [22] exist. Having the same limitation in using the out-of-date phenotype similarity network as in [19], study [20] prioritized protein complexes implicated in human diseases using a maximum information flow algorithm on a heterogeneous network which was constructed by combining a protein interaction network and the phenotypic similarity network. Recently, we also introduced a method for identification of disease associated protein complexes using a random walk with restart (Shortly called RWR) algorithm, which yields a global similarity measure, on a constructed functional similarity protein complex network. This method achieved relative high performance [21]. However, the functional similarity between protein complexes in the constructed protein complex network was only based on their shared protein elements.

In this study, we propose a novel method to identify novel disease-protein complex associations. First, we presented a framework to construct functional similarity protein complex networks where each node is a protein complex and two protein complexes are functionally connected if they either share protein elements, share annotating gene ontology (GO) [24] term or are connected by protein interactions. Then, we proposed a novel neighborhood-based algorithm (Shortly called NBH), which yields a local similarity measure, to prioritize disease candidate protein complexes. We compared the performance of our algorithm to two state-of-the-art network propagation algorithms, RWR [21] and PRINCE [14], on the three constructed functional similarity protein complex networks. The performance of each algorithm was assessed based on a set of known disease-protein complex associations using a leave-one-out cross validation method. The comparative results showed that NBH statistically significantly outperformed that of the RWR and PRINCE algorithms in predicting novel disease-protein complex associations. In addition, NBH consumed less running time in ranking candidate protein complexes than that of the RWR and PRINCE algorithms. Moreover, relatively high performances achieved for all the constructed functional similarity protein interaction networks and the three algorithms indicated the stability and feasibility of our proposed method for the identification of novel disease associated protein complexes. Furthermore, a case study on prostate cancer was performed. As a result, 69 out of top 100 highly ranked protein complexes were shown to be associated with prostate cancer.

## Methods

### Databases of protein complexes and disease phenotype-gene associations

First, we obtained 1,704 human protein complexes from a database of mammalian organisms protein complex CORUM [5] (See in Additional file 1: Table S1). These protein complexes were manually annotated with their functions, localization, subunit composition and literature references. Then, we used known disease phenotype-gene associations from OMIM [25] to construct known disease phenotype-protein complex associations.

### Construction of known disease phenotype-protein complex associations

To our knowledge, there is no standard database of disease-protein complex associations in public resources. Therefore, based on an observation from a number of studies that mutations in multiple proteins that form a protein complex may lead to the same disease [7,8,26-29], we defined a known disease phenotype-protein complex association as follows: a protein complex is associated with a disease phenotype if at least one of its protein elements is associated with the disease phenotype. Based on this definition of the association, the set of collected human protein complexes and the set of known disease phenotype-gene associations, we constructed 282 disease phenotypes and their known associated protein complexes (See in Additional file 1: Table S2).

### Construction of functional similarity protein complex networks

**Figure 1:**
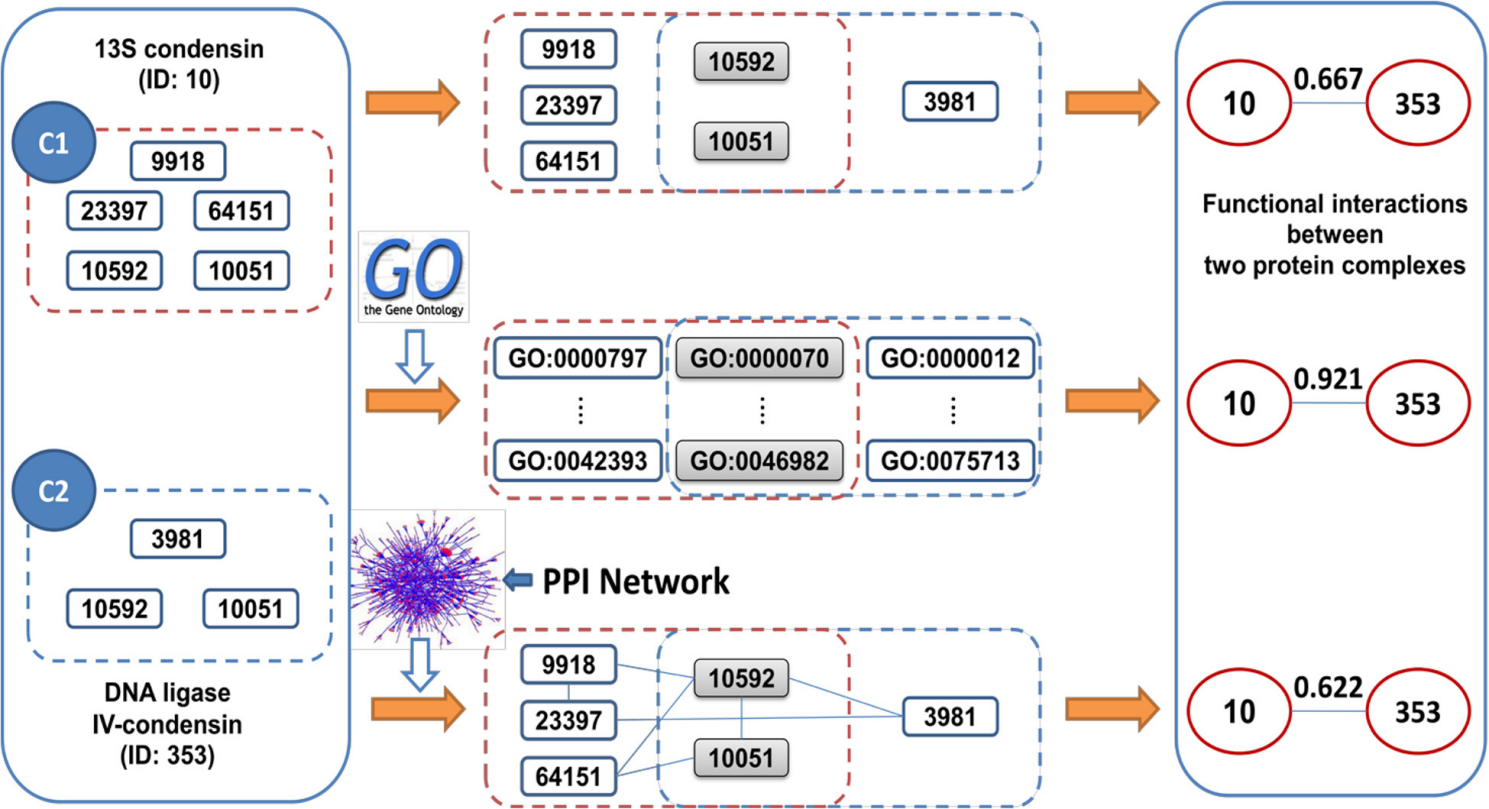
An illustrative example of construction of functional interactions between two protein complexes. Protein complexes *13S condensing* (ID: 10) and *DNA ligase IV-condensin* (ID: 353) compose of five and three protein elements, respectively. Functional interactions between these two protein complexes are specified based on shared protein elements, shared annotating GO terms, or protein interactions.

A protein complex is formed by structurally and functionally related protein elements. Indeed, protein elements within a protein complex have higher GO-based semantic similarities than that on all proteins [30]. In addition, protein complexes often correspond to functionally and structurally cohesive substructures/densely connected regions in protein interaction network [31,32]. Therefore, to construct functional similarity protein complex networks where each node is a protein complex, we defined a functional similarity interaction between two protein complexes based on: i) shared protein elements; ii) shared annotating GO terms; and iii) protein interactions. Figure 1 shows an illustrative example of the construction of functional similarity interactions between two protein complexes.

Given two protein complexes *Ci* = {*p*_*k*_|*k* = 1…m} and *Cj* = {*p*_*l*_|*l* = 1…n}, where *p*_*k*_, *p*_*l*_ are protein elements of *Ci* and *Cj*, *m* and *n* are the number of protein elements belonging to *Ci* and *Cj*, respectively, we here defined three kinds of functional similarity interactions between *Ci* and *Cj* based on shared protein elements, shared annotating GO terms and protein interactions.

#### A functional similarity interaction between two protein complexes based on their shared protein elements

A protein complex is formed by functionally related protein elements. Therefore, it could be accepted that the more number of protein elements two protein complexes share, the more functionally related they are. Indeed, it was shown that this kind of interaction not only reflects physical interaction of complexes, but may also represent common regulation, localization, turnover or architecture [33]. Therefore, as we did in our previous study [21], we defined a functional similarity interaction between two protein complexes based on their shared protein elements as follows: Two protein complexes are functionally interacted with each other if they share at least one protein element. And, a weight of this interaction is number of the shared protein elements normalized by number of elements of a protein complex which has a smaller number of elements. It is formally defined as follows:

$$ {S}_C=\frac{\left| Ci\cap Cj\right|}{min\left(\left| Ci\right|,\ \left|Cj\right|\right)} $$

Based on this definition and the set of collected human protein complexes, we constructed a functional similarity protein complex network including 1,579 nodes and 16,981 interactions (shortly called *SharedProteinComNet*).

#### A functional similarity interaction between two protein complexes based on their shared annotating GO terms

Functions of each protein elements are described by annotating GO terms. Therefore, we additionally constructed a functional similarity interaction between two protein complexes based on shared annotating GO terms, where a list of genes and annotating GO terms were collected from NCBI Entrez Gene FTP site (ftp://ftp.ncbi.nlm.nih.gov/gene/DATA/gene2go.gz). More specifically, we defined the interaction based on a measure of gene functional similarity introduced in [34] as follows: Two protein complexes are functionally interacted with each other if they share at least one annotating GO term, where annotating GO terms include direct annotating GO terms and their ancestors in the GO directed acyclic graph. Then, weight of the interaction is defined as number of the shared annotating GO terms normalized by number of annotating GO terms of a protein complex whose protein elements were annotated with smaller number of annotating GO terms. Formally, a functional similarity interaction between *Ci* and *Cj* is defined based on their shared annotating GO terms as follows:

$$ {S}_C=\frac{\left|G{O}_{Ci}\cap G{O}_{Cj}\right|}{min\left(\left|G{O}_{Ci}\right|,\ \left|G{O}_{Cj}\right|\right)} $$

where *GO*_*Ci*_, *GO*_*Cj*_ are sets of GO terms annotating to protein elements in *Ci* and *Cj*, respectively.

As in [34], *GO*_*Ci*_ and *GO*_*Cj*_ are defined as follows:

$$ G{O}_{Ci}={\displaystyle \underset{k}{\overset{m}{\cup }}}\left(G{O}_k\cup anc\left(G{O}_k\right)\right) $$

And

$$ G{O}_{Cj}={\displaystyle \underset{l}{\overset{n}{\cup }}}\left(G{O}_l\cup anc\left(G{O}_l\right)\right) $$

where: *GO*_*k*_ and *GO*_*l*_ are set of direct annotating GO terms of protein *p*_*k*_ and *p*_*l*_, respectively; *anc*(*GO*_*k*_) and *anc*(*GO*_*l*_) are ancestors of *GO*_*k*_ and *GO*_*l*_ excluding root terms (i.e., GO:0008150 for biological process, GO:0005575 for cellular component and GO:0003674 for molecular function) in GO directed acyclic graph of each sub-category, respectively.

For each GO sub-category, we constructed a functional similarity protein complex network. Then we integrated them using “per-edge average” method as follows:

$$ {\overline{S}}_C=\frac{1}{M}{\displaystyle \sum_{k=1}^M}{\left({S}_C\right)}_k $$

where *M* is number of networks containing interaction between protein complex *C*_*i*_ and *C*_*j*_. (*S*_*C*_)_*k*_ is functional similarity interaction between *C*_*i*_ and *C*_*j*_ in network *k*.

Consequently, we constructed a functional similarity protein complex network including 1,683 nodes and 1,415,266 interactions based on annotating GO terms (shortly called *SharedGOTermComNet*).

#### A functional similarity interaction between two protein complexes based on their shared protein interactions

A past study proposed a method to measure a functional relationship between two gene sets based on protein interaction network [35]. By considering a protein complex a special case of a gene set, the study showed that protein complexes with high functional similarities tend to be involved in the same functional catalogue and these functional similarities were successfully used in prioritizing candidate cancer-associated protein complexes [35]. In this study, we additionally used that method to define a functional similarity interaction between two protein complexes based on protein interaction network. To this end, we collected a human physical protein interaction network (PPI) consisting of 10,486 genes and 50,791 interactions from the NCBI Entrez Gene FTP site (ftp://ftp.ncbi.nlm.nih.gov/gene/GeneRIF/interactions.gz). This network was constructed by integrating BIND [36], BioGRID [37] and HPRD [38]. Formally, a functional similarity interaction between *Ci* and *Cj* was defined based on protein interactions among protein elements belonging to the two protein complexes as follows:

$$ {S}_C=\frac{{\displaystyle {\sum}_{k=1}^m}{\displaystyle {\sum}_l^n}\frac{1}{SP\left({p}_k,\ {p}_l\right)}}{m\times n} $$

Where

$$ SP\left({p}_k,\ {p}_l\right) = \left\{\begin{array}{c}\hfill 1\kern5em  if\ {p}_k\equiv {p}_l\  or\ {p}_k,\kern0.5em {p}_l\in {C}_i\cap {C}_j\hfill \\ {}\hfill Length\  of\  shortest\  path\kern0.5em  between\ {p}_k\  and\ {p}_l\hfill \end{array}\right. $$

Based on this definition, the set of collected human protein complexes and the human physical protein interaction network, we constructed a functional similarity protein complex network including 1,681 nodes and 1,412,040 interactions based on protein interactions (shortly called *SharedPPIComNet*).

### Network-based ranking algorithms

Given a connected weighted graph G(*V*, *E*) with a set of nodes *V* = {*v*_*1*_*, v*_*2*_*, …, v*_*N*_} and a set of links *E* = {(*v*_*i*_*, v*_*j*_)| *v*_*i*_*, v*_*j*_∈*V*}, a set of source nodes *S* ⊆ *V* and a *N* × *N* adjacency matrix *W* of link weights*.* Here, we are going to introduce our proposed neighborhood-based algorithm. In addition, we also briefly describe the RWR algorithm, which was used in our previous study [21], and the PRINCE algorithm [14]. These three algorithms will be used for measuring relative similarity of node *v*_*i*_ to *S*. By modelling a functional similarity protein complex network as a graph (i.e., nodes present protein complexes, links present functional interactions among protein complexes, *W* presents pair-wise similarities between protein complexes, and *S* presents known disease-associated protein complexes), a ranking of candidate protein complexes based on their relative similarity to *S* is to predict novel disease-associated protein complexes. The relative similarity also measures how relevant to a disease a candidate protein complex is.

#### The proposed neighborhood-based algorithm

The neighborhood-based algorithm (shortly called NBH) was based on direct neighbors of source nodes (*S*). Formally, the relative similarity of a node *v*_*i*_ to a set of source nodes (*S*) was defined as following:

$$ {p}_i={\displaystyle \sum_{j\in S}}{w}_{ij} $$

where *w*_*ij*_ is weight of link (*v*_*i*_, *v*_*j*_). This score is 0 for nodes not connected to any source nodes.

#### Random Walk with Restart (RWR)

RWR is a variant of the random walk [39] and it mimics a walker that moves from a current node to a randomly selected adjacent node or goes back to source nodes with a back-probability *γ* ∈(0, 1). RWR can be formally described as follows:

$$ {P}^{t+1}=\left(1-\gamma \right)W\mathit{\hbox{'}}{P}^t+\gamma {P}^0 $$

where *P*^t^ is a *N* × *1* probability vector of |*V*| nodes at a time step *t* of which the *i*th element represents the probability of the walker being at node *v*_*i*_∈*V*, and *P*^*0*^ is the *N* × *1* initial probability vector where the value of an element corresponding to a non-source node or a source node is zero or 1/|*S*|, respectively. The matrix *W’* is represented by a transition probability matrix and thus (*W’*)_*ij*_, the (*i*, *j*) element in *W’*, denotes a probability with which a walker at *v*_*i*_ moves to *v*_*j*_ among *V*\{*v*_*i*_}. Formally, it is defined as follows:

$$ {\left(W\mathit{\hbox{'}}\right)}_{ij}=\frac{(W)_{ij}}{{\displaystyle {\sum}_{k\in {\left({V}_{out}\right)}_i}}{(W)}_{ik}} $$

where (*V*_*out*_)_*i*_ is a set of outgoing nodes of *v*_*i*_*.*

All nodes in the network are eventually ranked according to the steady-state probability vector *P*^*∞*^, which is obtained by repeating the iterations until ||*P*^*t+1*^-*P*^*t*^|| < 10^−6^ in this study.

#### PRINCE

Study [14] introduced PRINCE for disease gene prediction. Similar to RWR, PRINCE is a kind of network propagation algorithm, it simulates a process where nodes for which prior information exists pump information to their neighbors though an iteration process. At each iteration, every node propagates the information received at the previous iteration to its neighbors. PRINCE can be formally described as follows:

$$ {P}^{t+1}=\alpha W\mathit{\hbox{'}}{P}^t+\left(1-\alpha \right){P}^0 $$

where *P*^*0*^ represents a prior knowledge function. Similar to RWR, in this study, it is the *N* × *1* initial probability vector where the value of an element corresponding to a non-source node or a source node is zero or 1/|*S*|, respectively. The first part of the equation represents the smooth propagation process which assigns similar values to adjacent nodes, while the second part represents prior knowledge. Trade-off parameter *α* ∈(0, 1) weighs the relative importance of these constraints with respect to one another. Different from RWR, The matrix *W’* is represented by a row-normalized matrix and defined formally as follow:

$$ {\left(W\mathit{\hbox{'}}\right)}_{ij}=\frac{(W)_{ij}}{\sqrt{{\displaystyle {\sum}_{k\in {\left({V}_{in}\right)}_i}}{(W)}_{ki}\times {\displaystyle {\sum}_{l\in {\left({V}_{in}\right)}_j}}{(W)}_{lj}}} $$

where (*V*_*in*_)_*i*_ and (*V*_*in*_)_*j*_ are a set of incoming nodes of *v*_*i*_ and *v*_*j*_*,* respectively.

Similarly to the RWR algorithm, all nodes in the network are eventually ranked according to the steady-state probability vector *P*^*∞*^, which is obtained by repeating the iterations until ||*P*^*t+1*^-*P*^*t*^|| < 10^−6^ in this study.

### Performance evaluation

Ranking performance was assessed through the leave-one-out cross-validation (Shortly called LOOCV) process. Let us assume that a functional similarity protein complex network *G*(*V*, *E*), a set of known disease-associated protein complexes (*D* ⊆ *V*) and a set of candidate protein complexes (*C*) are given. A protein complex *s*∈*D* was held out for validation and the remaining known disease-associated protein complexes were specified to a set of source nodes (*i.e.*, *S* = *D*\{*s*}). The network-based ranking algorithms were used to prioritize all the candidate protein complexes. This process was repeated by setting every *s*∈*D* to a held-out protein complex. For a reliable performance comparison, we drew the receiver operating characteristic (ROC) curves and computed the area under the curve (AUC) based on the rank of held-out protein complex *s* and candidate protein complexes in set *C*∪{*s*}. More specifically, given a threshold *τ*, we counted *TP* (true positives), *FN* (false negatives), *FP* (false positives), and *TN* (true negatives), which were formally defined as following:

$$ TP={\displaystyle \sum_{s\mathit{\in}D}}I\left( rank(s)\le \tau \right)\kern4.5em FN={\displaystyle \sum_{s\mathit{\in}D}}I\left( rank(s)>\tau \right) $$

$$ FP={\displaystyle \sum_{c\mathit{\in}C}}I\left( rank(c)\le \tau \right)\kern4.5em TN={\displaystyle \sum_{c\mathit{\in}C}}I\left( rank(c)>\tau \right) $$

where *rank*(*s*), *rank*(*c*) and *I*(∙) denote the rank of *s*, the rank of a candidate protein complex *c* out of the set *C*∪{*s*} and the indicator function, respectively. Then, we defined *sensitivity* and (1-*specificity*) as follows:

$$ sensitivity=\frac{TP}{TP+FN}\kern4.5em 1\mathit{\hbox{-}} specificity=\frac{FP}{FP+TN} $$

By varying *τ* from one to the number of protein complexes in the set *C*∪{*s*}, the relationship between *sensitivity* and (1-*specificity*) was plotted. The ROC curve is the curve constructed based on those pairs of values, and the AUC is the area under the ROC curve. In this study, we considered candidate protein complexes set as all protein complexes that are not known to be associated with the disease (*i.e.*, *V*\*D*) in *G*.

## Results and discussion

### Performance comparison

**Figure 2:**
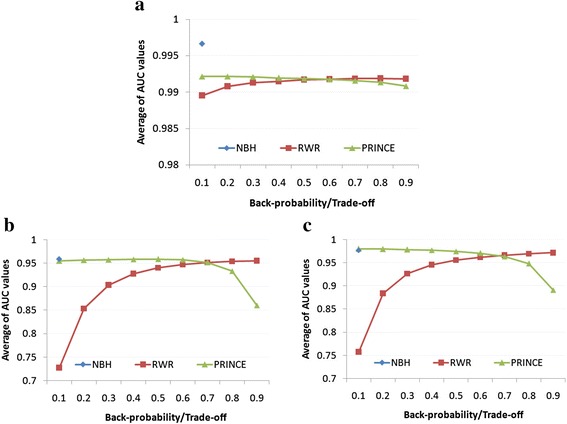
Performance comparison between network-based ranking algorithms on different functional similarity protein complex networks. **(a)** *SharedProteinComNet*. **(b)** *SharedGOTermComNet*. **(c)** *SharedPPIComNet*. For RWR and PRINCE, back-probability and trade-off parameters were varied in a range of [0.1, 0.9], respectively. Vertical axis represents average AUC values over 270 disease phenotypes.

Due to using LOOCV method, we collected only 270 disease phenotypes which are known to be associated with at least two protein complexes to assess the performance of each algorithm. For RWR and PRINCE algorithm, we varied back-probability and trade-off parameters respectively in a range of [0.1, 0.9]. The AUC values were calculated for each disease phenotype on each individual functional similarity protein complex network. Then, the performance of each algorithm was averaged over those disease phenotypes for each individual functional similarity protein complex network. Figure 2 shows that the performance of NBH was statistically significantly better than that of RWR and PRINCE (i.e., All P-values < 0.05 using two sample t-Test. See more detail in Table 1).

**Table 1:** **Performance comparison between network-based ranking algorithms on different functional similarity protein complex networks**

**#**	**Protein complex network**	**NBH**	**RWR**	**PRINCE**
*1*	*SharedProteinComNet*	0.99665 (±0.00988)	0.99134 (±0.01663)	0.99174 (±0.01633)
			(P-value = 4.37 × 10^−14^)	(P-value = 1.7 × 10^−12^)
*2*	*SharedGOTermComNet*	0.95869 (±0.07599)	0.90671 (±0.10804)	0.94305 (±0.08432)
			(P-value = 1.28 × 10^−27^)	(P-value = 8.2 × 10^−4^)
*3*	*SharedPPIComNet*	0.97704 (±0.05926)	0.92646 (±0.12794)	0.96257 (±0.08166)
			(P-value = 1.28 × 10^−27^)	(P-value = 1.51 × 10^−4^)

–P-values represent the statistical significance of performance comparison between NBH and RWR/PRINCE algorithms. They were calculated using two-sample t-Test for mean assuming unequal variances.

–Data in each cell represent mean (±standard deviation).

–Performance of NBH is statistically significantly better than that of RWR and PRINCE for all three functional similarity protein complex networks. Data row #1, #2 and #3 are detail performance comparison of the three algorithms on *SharedProteinComNet*, *SharedGOTermComNet* and *SharedPPIComNet* networks corresponding to Figure 2(a), (b) and (c), respectively.

Another advance of NBH compared to RWR and PRINCE is that NBH is free of parameters, whereas the performance of RWR and PRINCE is controlled by back-probability and trade-off parameters, respectively. Indeed, Figure 2 shows that the performance of RWR was improved when back-probability increases (i.e., slopes of regression lines for RWR in Figure 2(a), (b) and (c) are 0.00230, 0.22108 and 0.20141 with P-values are 0.00307, 0.00409, 0.005 using ANOVA test for regression, respectively). In contrast, the performance of PRINCE declined when trade-off increases (i.e., slopes of regression lines for PRINCE in Figure 2(a), (b) and (c) are −0.00147, −0.03838 and −0.08100 with P-values are 0.00015, 0.02758, 0.00714 using ANOVA test for regression, respectively). This opposite trends of RWR and PRINCE is because RWR and PRINCE algorithms are generally similar in that the first part of RWR and PRINCE represents the smooth propagation process which assigns similar values to adjacent nodes, while the second part represents prior knowledge. However, the parameters affect inversely for each algorithm (i.e., when back-probability increases the random walker of RWR tend to go back to the source nodes and therefore give higher score for nodes nearby source nodes. In contrast, when trade-off increases the random walker of PRINCE tends to go far from source nodes and therefore assign lower score for nodes nearby source nodes). Figure 2 also shows that RWR and PRINCE achieved best performance when back-probability and trade-off parameters are set to 0.9 and 0.1, respectively. This observation implied that by linking protein complexes by functional interactions, protein complexes associated with the same or similar disease phenotypes tend to be connected closely. This is also the reason why NBH, which is only based on neighbors of known disease protein complexes, achieved better performance than that of RWR and PRINCE.

**Figure 3:**
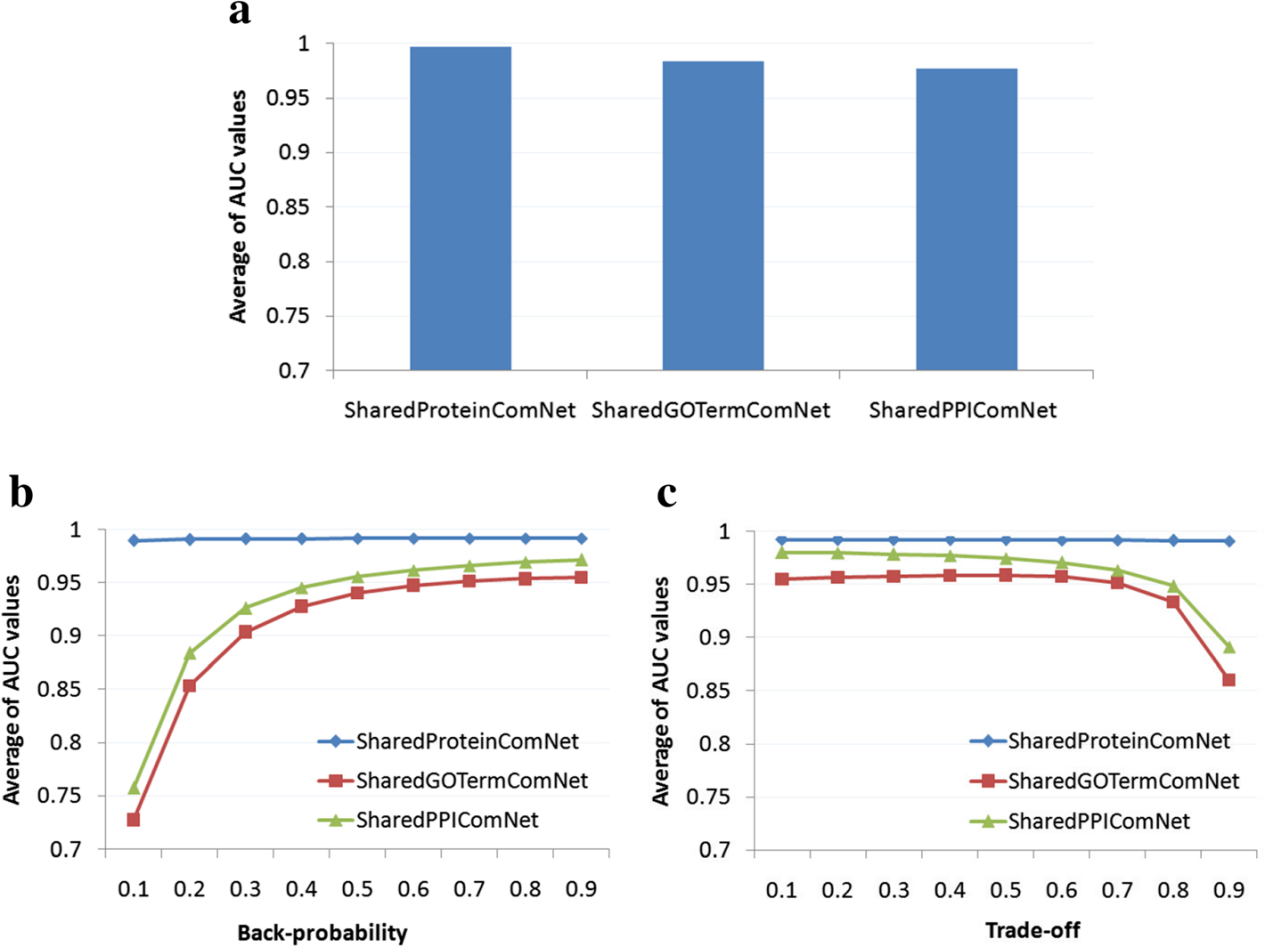
Performance comparison between functional similarity protein complex networks on different network-based ranking algorithms. **(a)** NBH. **(b)** RWR. **(c)** PRINCE. For RWR and PRINCE, back-probability and trade-off parameters were varied in a range of [0.1, 0.9], respectively. Vertical axis represents average AUC values over 270 disease phenotypes.

In addition, we observed that the performance of RWR shows less positive trend in *SharedProteinComNet* than that in *SharedGOTermComNet* and *SharedPPIComNet* (i.e., the slopes of regression lines are 0.00230, 0.22108 and 0.20141, respectively), and PRINCE was shown less as a negative trend in *SharedProteinComNet* than that in *SharedGOTermComNet* and *SharedPPIComNet* (i.e., the slopes of regression lines are −0.00147, −0.03838 and −0.08100, respectively). Considering the network density of *SharedProteinComNet* was much lower than that in *SharedGOTermComNet* and *SharedPPIComNet* (i.e., the network densities are 0.014, 0.999 and 1, respectively), this observation indicated that disease protein complexes are more connected in *SharedGOTermComNet* and *SharedPPIComNet*. However, networks with very high densities such as *SharedGOTermComNet* and *SharedPPIComNet*, may contain unreliable functional interactions that can reduce the performance of the methods. Indeed, Figure 3 shows that the performance of all algorithms on *SharedProteinComNet* was better than that on *SharedGOTermComNet* and *SharedPPIComNet* (i.e., All P-values < 0.05. See more detail in Table 2).

**Table 2:** **Performance comparison between functional similarity protein complex networks on different network-based ranking algorithms**

**#**	**Algorithm**	**SharedProteinComNet**	**SharedGOTermComNet**	**SharedPPIComNet**
*1*	*NBH*	0.99665 (±0.00988)	0.95869 (±0.07599)	0.97704 (±0.05926)
			(P-value = 6.74 × 10^−15^)	(P-value = 8.43 × 10^−8^)
*2*	*RWR*	0.99134 (±0.01663)	0.90671 (±0.10804)	0.92646 (±0.12794)
			(P-value = 1.06 × 10^−252^)	(P-value = 7.61 × 10^−122^)
*3*	*PRINCE*	0.99174 (±0.01633)	0.94305 (±0.08432)	0.96257 (±0.08166)
			(P-value = 7.14 × 10^−151^)	(P-value = 1.14 × 10^−63^)

–P-values represent the statistical significance of performance comparison between *SharedProteinComNet* and *SharedGOTermComNet*/*SharedPPIComNet* networks. They were calculated using two-sample t-Test for mean assuming unequal variances.

–Data in each cell represent mean (±standard deviation).

**–**Performance of *SharedProteinComNet* is statistically significantly better than that of *SharedGOTermComNet* and *SharedPPIComNet* for all three network-based ranking algorithms. Data row #1, #2 and #3 are detail performance comparison of the three functional similarity protein complex networks by NBH, RWR and PRINCE algorithms corresponding to Figure 3(a), (b) and (c), respectively.

**Figure 4:**
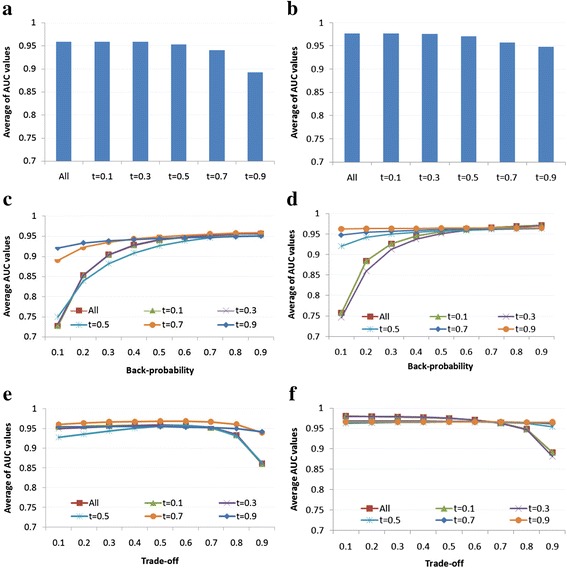
Investigation of the performance of each method on different functional similarity protein complex networks extracted with different thresholds (t). **(a)**, **(c)** and **(e)** are the performance of NBH, RWR and PRINCE for networks which are extracted from *SharedGOTermComNet*, respectively; **(b)**, **(d)** and **(f)** are the performance of NBH, RWR and PRINCE for networks which are extracted from *SharedPPIComNet*, respectively. For RWR and PRINCE, back-probability and trade-off parameters were varied in a range of [0.1, 0.9], respectively. Vertical axis represents average AUC values over 270 disease phenotypes. “All” is the original functional similarity protein complex networks (i.e., *SharedGOTermComNet* in **(a)**, **(c)** and **(e)**; *SharedPPIComNet* in **(b)**, **(d)** and **(f)**).

To test the hypothesis of whether density affect the performance of the algorithms, we varied a threshold (t) of interaction weight to extract different functional similarity protein complex networks of which interactions having weight no less than t. Particularly, for *SharedGOTermComNet* and *SharedPPIComNet*, we additionally extracted five networks corresponding to t = 0.1, 0.3, 0.5, 0.7, and 0.9. Figure 4 shows the performance of each algorithm on *SharedGOTermComNet* and *SharedPPIComNet* with different thresholds. As observed, for NBH (Figure 4(a) and (b)), the best performance was achieved with “All” (i.e., *SharedGOTermComNet* and *SharedPPIComNet*) and decreased when the threshold increased. However, for RWR (Figure 4(c) and (d)) and PRINCE (Figure 4(e) and (f)), it performed best and most stable with t = 0.7 or t = 0.9. This result indicated these two algorithms perform better when unreliable functional interactions were eliminated.

In addition to comparison of prediction performance of the proposed algorithm with RWR and PRINCE, we here show its advance in running time. To this end, we ran each method for 282 disease phenotypes, where their known disease associated protein complexes were used as source nodes. For RWR and PRINCE, we also varied back-probability and trade-off parameters in a range of [0.1, 0.9] and took the average of running time over this range. The final running time for each disease phenotype was averaged over the set of 282 disease phenotypes. Table 3 shows the comparison on the running time of different algorithms. It was obvious that our proposed algorithm run faster than both RWR and PRINCE about 32 times averaging on all the three functional similarity protein complex networks.

**Table 3:** **Running time (millisecond) comparison between network-based ranking algorithms on different functional similarity protein complex networks**

	**NBH (A)**	**RWR (B)**	**PRINCE (C)**	**Speedup (B/A)**	**Speedup (C/A)**
*SharedProteinComNet*	2	79	81	41.8	42.6
*SharedGOTermComNet*	110	3369	3428	30.6	31.2
*SharedPPIComNet*	115	2544	2497	22.2	21.8
**Average**				**31.5**	**31.9**

–Platform: Intel Core i3 3240 CPU 3.4GHz, 4GB RAM.

–Average running time for ranking all candidate protein complexes on functional similarity protein complex networks of a disease phenotype.

To our knowledge, only a few studies have directly been proposed for identification of disease-associated protein complexes [19-22]. In which, as showed in the previous section, NBH algorithm outperformed RWR, which was used in our previous study [21], in both prediction performance and running time for all the three constructed functional similarity protein complex networks. Meanwhile, the study [22] was specifically proposed to identify cancer-associated protein complexes based on gene expression data and it did not show the over prediction performance. For the two remaining studies, they used network propagation [19] and a maximum information flow [20] algorithms on complicated heterogeneous networks. For instance, study [19] constructed a heterogeneous network including three layers including a disease phenotype similarity network layer, a tissue-specific protein interaction network layer and a protein complex membership layer, then they applied the RWR algorithm on this heterogeneous network. Besides using an out-of-date disease phenotype similarity network collected from literature [23], this study can apply to only disease phenotypes of which a tissue-specific protein interaction network exist. Similarly, in the study [20], they also constructed a heterogeneous network including a disease phenotype similarity network layer and protein interaction network layer, in which the disease phenotype similarity network was also collected from literature [23]. It is obvious that, these two methods based on networks that are different from ours. Therefore, it is unfeasible to compare directly the performance of our proposed method with them. However, the best reported performances of these methods are inferior to ours (i.e., the performance in term of AUC value is about 0.88 and 0.92 in the study [19] and study [20], respectively; whereas the worst case for NBH is about 0.96 (See Table 1)).

### Case study: prostate cancer

Prostate cancer (MIM ID: 176807) is a complex disease and there were 22 genes associated with it as published in OMIM [25]. Following the definition of a known disease phenotype-protein complex association (See Methods section), we found that 12 protein complexes were known to be associated with prostate cancer (See in Additional file 1: Table S2). These associated protein complexes were set as source nodes and all others as candidates in *SharedProteinComNet*. After applying NBH to rank all candidates, we selected 100 highly ranked candidate protein complexes. By searching associations between 219 genes coding proteins involved in those selected protein complexes with prostate cancer on GeneRIF [40] (ftp://ftp.ncbi.nih.gov/gene/GeneRIF/generifs_basic.gz), we found 28 of them reported to be associated with prostate cancer (See in Additional file 1: Table S3). These protein-coding genes are involved in 69 protein complexes in the top 100 selected protein complexes. For instance, overexpression of Skp2 in protein complex “Ubiquitin E3 ligase (SKP1A, SKP2, CUL1, RBX1)” (ID: 1051) is associated with recurrence following radical prostatectomy in prostate cancer [41]. In addition, lower levels of nuclear beta-catenin in protein complex “JUN-TCF4-CTNNB1 complex” (ID: 1816) is associated with prostate cancer progression [42]. Elevated BRCA1 and NBN truncating mutation in protein complex “BRCA1-RAD50-MRE11-NBS1 complex” (ID: 202) are associated with prostate cancer [43,44] . Beside the 69 evidenced protein complexes, others in the top 100 ranked protein complexes can be candidates for future validation (See in Additional file 1: Table S4).

To make the results for identification of novel prostate cancer-associated protein complexes more convincing, we additionally randomly selected 200 sets of 100 protein complexes among protein complexes in the same functional similarity protein complex network. Then, we repeated the same procedure as we did for the 100 highly ranked candidate protein complexes to find novel prostate cancer-associated protein complexes for each set. The result showed that, on average, about 28 protein complexes in each set were enriched with genes which are associated with Prostate cancer (See in Additional file 1: Table S5). Therefore, the set of 100 highly ranked candidate protein complexes has more prostate cancer associations than expected by chance (P-values = 2.9 × 10^−200^, using one-sample t-Test).

## Conclusions

Protein complexes play important roles in cellular functions; however, their roles in causing disease have not been paid enough attention because there have been a few studies directly focusing on identifying disease-protein complex associations. In this study, we have proposed a novel method for identification of novel disease associated protein complexes. Comparing to our previous study [21], which used RWR algorithm on a functional similarity protein complex network built based on shared protein elements, in this study, we presented a framework to construct functional similarity protein complex networks based on not only shared protein elements, but also shared annotating GO terms and protein interactions. In addition, we proposed a novel neighborhood-based algorithm to prioritize candidate disease protein complexes. Comparing the performance of our proposed algorithm with that of the two state-of-the-art algorithms (i.e., RWR and PRINCE, which yields global similarity measure), we found that our proposed algorithm outperformed these two algorithms for all the three constructed functional similarity protein complex networks. In addition, it also consumed less running time than these algorithms in ranking candidate protein complexes for each disease phenotype. Moreover, our proposed algorithm is free of parameters; meanwhile the performance of RWR and PRINCE is controlled by back-probability and trade-off parameters, respectively. Comparing the performance of our proposed method with that of other methods also proposed for identification of disease associated protein complexes, we found that our proposed method is simpler since it is only based on homogeneous network of protein complexes (i.e., the three functional similarity protein complex networks only contain protein complexes as nodes), meanwhile other methods were based on a complicated heterogeneous networks in which a node can be either protein, protein complex or disease phenotype. In addition, the best reported performances of these methods are inferior to our proposed method. Finally, using the proposed method to identify novel prostate cancer associated protein complexes; we found that 69 out of top 100 highly ranked candidate protein complexes have evidences about the association with prostate cancer from literature.

## Additional file

Additional file 1: Table S1. List of 1,704 collected human protein complexes. **Table S2.** List of 282 disease phenotypes and their known associated protein complexes. **Table S3.** List of 28 protein coding genes (involved in 100 highly ranked candidate protein complexes) having evidences about the association with prostate cancer from literature. **Table S4.** List of 100 highly ranked candidate protein complexes. 69 out of them have evidences about the association with prostate cancer from literature. **Table S5.** List of protein complexes having evidences about the association with prostate cancer from literature in 200 randomly selected sets of 100 protein complexes.
